# The Psychological Impact on Parents of Children who Receive an Inconclusive Diagnosis for Cystic Fibrosis following Newborn Screening: A Systematic Mini-Review

**DOI:** 10.3390/children11010093

**Published:** 2024-01-12

**Authors:** Ioanna Loukou, Maria Moustaki, Konstantinos Douros

**Affiliations:** 1 Cystic Fibrosis Department, Agia Sofia Children’s Hospital, Thivon 1, 11527 Athens, Greece; i.loukou@paidon.gr (I.L.); cfcenter@paidon.gr (M.M.); 2 Pediatric Allergy and Respiratory Unit, 3rd Department of Pediatrics, “Attikon” University Hospital, School of Medicine, National and Kapodistrian University of Athens, Rimini 1, 12462 Athens, Greece

**Keywords:** anxiety, cystic fibrosis, CF related metabolic syndrome (CRMS), CF screen positive inconclusive diagnosis (CFSPID), newborn screening, perceptions, psychological impact

## Abstract

Newborn screening (NBS) has been available for the diagnosis of cystic fibrosis (CF) over the last decades. Through the implementation of NBS, a new designation emerged, that of CF related metabolic syndrome (CRMS) or cystic fibrosis screen positive inconclusive diagnosis (CFSPID). As there is uncertainty regarding the clinical progression of these infants to CF, some studies have investigated the psychological impact of CRMS/CFSPID on their parents. This systematic narrative review aimed to describe the findings of the relevant studies. The number of studies is limited and the study samples are relatively small. It seems that there is a negative impact of CRMS/CFSPID on parental mental health. While some studies indicated similar levels of parental anxiety among those with infants diagnosed with CF and those with CRMS/CFSPID, not all studies reached the same conclusion. Parental uncertainty represents another mental dimension of the impact associated with the designation of CRMS/CFSPID. These observations suggest that parents of infants with CRMS/CFSPID should be provided with effective communication, and it may also be beneficial to consider parental mental screening. More robust and long-term studies are required to detect differences in parental emotional status between those with infants diagnosed with CF and those with CRMS/CFSPID.

## 1. Introduction

Newborn screening (NBS) has been available over the last decades for the early diagnosis of cystic fibrosis (CF). This screening was implemented in many countries worldwide. However, despite the benefits of early diagnosis of CF through the NBS program, several newborns remain with an uncertain diagnosis of CF. Since 2016 a harmonized definition was introduced in Europe and the USA and the designation of CF-related metabolic syndrome/CF screen-positive inconclusive diagnosis (CRMS/CFSPID) was used thereafter for the CF inconclusive diagnosis following newborn screening [1]. The composite term CRMS/CFSPID was applied to infants with either a normal sweat test and two CFTR variants with at least one with unclear phenotypic consequences or an intermediate sweat test and one or no CFTR-causing variants. A proportion of children with CRMS/CFSPID convert to CF or are reclassified as subjects with CF [1]. While there are updated guidelines for the optimal approach to the evaluation and management of this population, there are still unresolved issues that require attention. The psychological impact on parents of infants with CRMS/CFSPID deserves attention, given the designation’s inherent uncertainty regarding the health status of their infants [2]. It is noteworthy that the diagnosis of CF brings the disease to the forefront for parents, even when their child is still an infant and may be asymptomatic [3]. Recognizing this, studies have been conducted to assess the psychosocial consequences, not only of typical CF but also of inconclusive CF diagnoses.

The aim of this systematic narrative review was to present the findings of studies evaluating the psychosocial impact on parents of infants with inconclusive CF diagnoses. The goal is to facilitate the improvement of communication strategies in pediatricians caring for these infants.

## 2. Methods

For the purpose of this review, the PubMed database was searched using the terms “cystic fibrosis newborn screening OR CFSPID OR CRMS OR cystic fibrosis inconclusive diagnosis OR intermediate cystic fibrosis” AND “anxiety OR psychosocial or psychological OR impact OR perceptions”. The selection of the studies was performed following the Preferred Reporting Items for Systematic reviews and Meta-Analyses (PRISMA) guidelines [4]. The search was limited to articles in the English language, conducted from PubMed inception until 10 December 2023, resulting in the identification of 354 articles. Data were extracted by two researchers, who reviewed the search results independently. Any disagreements were resolved by consensus and discussion.

**Figure 1:**
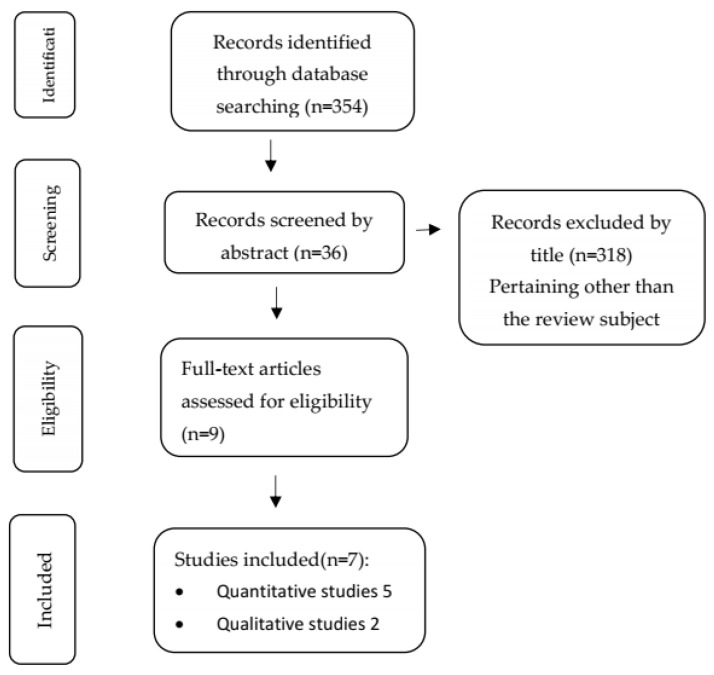
Diagram of the literature search process.

Following title screening, 36 articles were retained. The remaining 318 that were excluded by title were not relevant to the subject of this review or they were review articles. From the abstracts that were then screened 25 were excluded as irrelevant to the subject of this review and 9 articles were selected. After a thorough review of the full-text manuscripts, 7 articles were deemed eligible as the other two did not relate to the subject. The whole process is depicted in Figure 1.

## 3. Results

### 3.1. Included Studies

The main characteristics of the studies included in the present review are presented in Table 1.

### 3.2. Data from Quantitative Studies

The first psychological analysis of families with children with an uncertain CF diagnosis was published in 2009 [5]. Three groups of families from Italy were compared in this study. The research team employed a structured questionnaire consisting of 25 items divided into three sections. The initial section primarily encompassed queries related to parental knowledge regarding routine neonatal screening tests and the parental response upon receiving information about ambiguous results. The second section, also administered to healthy controls, delved into the present parental perception of their child’s health, the level of parental anxiety regarding it and potential emotional disturbances. The third section investigated the impact of neonatal screening results on the parent–child relationship, marital dynamics and the parental stance on future family planning. The children were all screened by NBS for CF. The first group comprised children with an ambiguous diagnosis of CF, the second group included children diagnosed with CF and the third group consisted of healthy controls (screen-negative). Each group included 11 families. According to their findings, the communication of sweat test results caused anxiety to both parents of children with CF and to those with an uncertain diagnosis. However, anxiety levels were significantly lower among families with children with an uncertain CF diagnosis compared to those with a CF diagnosis. The three groups also differed regarding the perception of their child’s health. A significant difference was observed, with a high proportion of families with children diagnosed with CF perceiving their child’s health as poor compared to only a single family within the group with an uncertain diagnosis. However, this difference diminished over time, as the negative perception of families with children diagnosed with CF improved. The levels of parental anxiety about their children’s health were significantly higher among families with children with CF compared to those with children with an uncertain diagnosis or healthy controls. The above parameter was not different between healthy controls and those with an uncertain CF diagnosis. No difference was observed between families with children diagnosed with CF and those with an uncertain diagnosis of CF regarding the emotional disturbances they attributed to their child’s health. In contrast, a significant difference was noted when compared to the emotional well-being of healthy control families. However, when parental answers were stratified by gender, a significant difference persisted only for mothers and not for fathers. Overall, the above-mentioned findings indicated that even an uncertain CF diagnosis is stressful for families.

Eight years later, a similar study from Ontario was published with both quantitative and qualitative results [6]. The quantitative component comprised three validated psychological measures: (a) the State Subscale of the State-Trait Anxiety Inventory for parental anxiety, (b) a modified version of the Parental Perceptions of Uncertainty in Illness Scale for parental uncertainty about childhood health and (c) the Child Vulnerability Scale for maternal perception of the child’s vulnerability. These questionnaires were exclusively administered to mothers. Therefore, the quantitative component constituted three measures (a) maternal anxiety (b) maternal perception of uncertainty (c) maternal perception of the child’s vulnerability. The study sample comprised three groups, as in the previous study by Perobelli et al. [5]. There was no difference regarding the anxiety and vulnerability levels among the three groups. However, the levels of maternal uncertainty were significantly higher among mothers with children with an inconclusive diagnosis compared to mothers with children with CF or to those with healthy controls.

In contrast to these results, Tluczek et al., from the USA found that parents of children with either CF or inconclusive CF perceived their children as more vulnerable compared to healthy controls who screened negative [7]. They utilized validated questionnaires, including: (a) a short version of the State-Trait Anxiety Inventory for parental anxiety assessment, (b) the Center for Epidemiologic Studies-Depression Scale for parental depression evaluation, (c) the Child Vulnerability Scale for assessing parental perception of their child’s vulnerability to illness, (d) the Parent Protection Scale for evaluating parental protectiveness and (e) a single item for rating parental expectations regarding their children developing CF symptoms. However, the levels of depression and protectiveness towards the child were lower among parents with children with CF and inconclusive CF compared to healthy controls. These seemingly unexpected findings might be explained by the qualitative findings of the study, namely that these parents expressed hope for their children to lead “normal” lives (like being involved in sports, going to college and having a child someday) while remaining mindful of their special healthcare needs.

Another quantitative study from the USA compared the levels of maternal anxiety and depression between two groups which consisted of mothers with children with CF and mothers with children with a CRMS/CFSPID designation [8]. They employed validated questionnaires, including: (a) The Edinburgh Postpartum Depression Scale for maternal postpartum depression assessment, (b) The Patient Health Questionnaire for evaluating depression in children older than 12 months and (c) the Generalized Anxiety Disorder-7 for assessing anxiety in mothers of children older than 12 months. The researchers did not include healthy controls but they compared the anxiety and depression rates of two groups with those in the general population. They showed that there was no difference in any item of maternal mental health evaluation (postpartum depression, depression and anxiety) between the two groups of mothers. The rates of postpartum depression were 31% among mothers with children diagnosed with CF, 15% among mothers with children with CRMS/CFSPID and 11% among adult women in the general population. The depression rates were 40%, 40%, and 22%, whereas the anxiety rates were 37%, 27% and 19%, respectively. Using semi-structured questionnaires, they identified similar rates among mothers in both study groups who reported that the diagnosis of CF or CRMS/CFSPID impacted their responses to postpartum depression, as well as depression and anxiety measures. Overall, 78% of mothers in the CF group and 79% in the CRMS/CFSPID group perceived that their child’s diagnosis influenced their emotional health. Additionally, 68% of mothers who received genetic counseling reported a positive impact from the process, regardless of the study group to which they belonged.

A recent study from Italy utilized both quantitative and qualitative methodologies. The quantitative methodology featured three validated questionnaires: (a) the Impact of Event Scale Revised for assessing parental distress following traumatic events, (b) the Patient Health Questionnaire for evaluating parental depressive symptoms in the last two weeks and (c) the Generalized Anxiety Disorder-7 scale for screening parental generalized anxiety disorder. Through quantitative tools, the study revealed elevated levels of anxiety in parents of children with either a CF diagnosis or inconclusive CF, with no significant difference between the two groups. Additionally, both groups exhibited depressive symptoms, without a significant difference between them. The study did not include a control group [9].

### 3.3. Data from Qualitative Studies

The qualitative studies used data derived from answers to semi-structured interviews with open ended questions.

The study by Hayeems et al., from Canada incorporated data from parental interviews in addition to quantitative data [6]. Specifically, only parents of children with an inconclusive diagnosis were interviewed, while the quantitative arm of the study included parents of children with CF and parents of healthy controls as well. The authors demonstrated that uncertainty led some parents to develop an enhanced sense of medical vulnerability for their children with inconclusive CF, adopting, in turn, a somewhat over-protective attitude. They did not feel comfortable with the CRMS/CFSPID designation, as its meaning does not entirely clarify whether the child has atypical CF or is healthy. However, they admitted that they felt somehow relieved that their children did not have typical CF. While endorsing the value of periodic monitoring, some parents may become reluctant over time toward regular surveillance, fearing potential negative psychological impacts on their children.

The study of Tluczek et al., also incorporated qualitative data obtained from parents’ responses to three open-ended questions. Notably, parents expressed optimism about their child’s future. However, when asked to envision it, most parents in the intermediate CF classification group responded ‘normal,’ whereas parents in the healthy control group focused on career and academic issues [7].

Tosco et al., also incorporated qualitative information through interviews with both parents of children with CF and parents of children with inconclusive diagnoses. In line with their quantitative results, they found that a negative psychological impact was evident in both groups after receiving the diagnosis of CF or inconclusive CF. Initially, both groups considered their child normal, but following either diagnosis, they tended to view their child as more fragile. Interestingly, in a continuum drawing test, parents of children with CF perceived their children as healthy or slightly ill, while those with inconclusive diagnoses considered their children as healthy [9].

Two qualitative studies, conducted by Tluczek et al. [10] and Johnson et al. [11], respectively, explored the psychological impact of inconclusive CF diagnosis on parents. Tluczek et al. [10] described the experiences of ten parents with five infants facing an inconclusive CF diagnosis. Uncertainty was the primary aspect of parental experience in this study, stemming from the ambiguity of the diagnosis and the challenge of distinguishing common childhood problems from potential CF symptoms. The level of uncertainty appeared to be influenced by parental education status, with less educated parents reporting lower levels of uncertainty. Both mothers and fathers experienced distress, but mothers tended to express it more openly. Interestingly, expressions of parental distress lessened over time as the infants remained healthy. The study of Johnson et al. [11] also highlighted a negative psychological impact on parents of children with an inconclusive diagnosis. The parents experienced distress, primarily attributed to the uncertainty associated with the CRMS/CFSPID designation. Some parents leaned towards adhering to the CF label within this designation, emphasizing its prominence. The perception of their child’s health status was unclear and varied widely among parents. Responses ranged from those who believed their child had CF to those who perceived them as a healthy carrier.

## 4. Discussion

Many countries implement routine NBS programs for CF with the aim of diagnosing neonates before the manifestation of clinical symptoms and signs, allowing for earlier access to appropriate care. While the disease is not preventable, NBS has contributed to improved well-being and survival in adulthood [12]. Notably, the primary benefit is observed in nutritional status rather than respiratory disease outcomes [13]. The CDC and Cystic Fibrosis Foundation adopted the recommendation of NBS for CF taking into account its moderate benefits and the low risk of harm [12].

Regardless of the screening protocol followed, the process is sequential and involves multiple steps. The initial step of the screening process is the measurement of Immunoreactive Trypsinogen (IRT) during the newborn period. In the case of a positive test, parents are informed for further laboratory investigation, the specifics of which depend on the protocol in place. After completing the investigation, it is found that some of the initially positive tests are false positives, and the corresponding children are healthy. However, even false positive results can cause anxiety and distress in parents as they await the results of the subsequent sweat test [14,15]. While the majority are relieved when the sweat test falls within the normal range, a minority continues to think about the results often or even constantly, one year later [16].

In addition to a definitive CF diagnosis and false positive results, inconclusive CF diagnoses may arise through the implementation of NBS screening. This term serves as a designation rather than a firm diagnosis. While the majority of children with a CRMS/CFSPID designation will remain well, a proportion may eventually convert or be reclassified as having CF over time [17]. Consequently, it is reasonable to assume that communicating this designation to parents following a positive NBS test may cause distress and concerns. Importantly, these concerns may not attenuate over time, in contrast to the experience of the majority of parents of infants with false positive NBS test results.

The results of quantitative studies conducted to explore this issue indicated that anxiety, uncertainty, and emotional disturbances are the primary psychological consequences for parents of children designated with CRMS/CFSPID [5,6,8,9]. It is of importance that some quantitative studies that included for comparison parents of children with CF and parents of healthy controls showed that uncertainty and anxiety or depression was comparable among the CRMS/CFSPID group and the CF group [5,9]. This point is rather alarming as the prevalence of anxiety and depression in caregivers of patients with CF is rather high in general [18]. This may be attributed to parents perceiving the CRMS/CFSPID designation as a “full CF” diagnosis, leading to substantial distress. Uncertainty is not surprising given the unclear prognosis, although the majority of children, though not all, remain well.

The uncertainty among parents about the designation is not unexpected, given the unclear prognosis for these children. Even healthcare professionals specializing in CF are not definitively certain about the long-term outcomes, as more studies and registry data are needed. It is not surprising that parents find it difficult to understand the meaning of the CRMS/CFSPID designation. Some studies have even indicated that parents of carrier neonates may worry about their child’s health status [19].

Uncertainty also emerged as the primary finding regarding the parental psychological status when evaluating infants with the CRMS/CFSPID designation using qualitative tools [10]. This designation made parents reconsider their perception of the traditional medical model, which typically represents medicine as a certain shelter. The CRMS/CFSPID designation introduces an element of unknown long-term prognosis, challenging the conventional certainty associated with medical care [10]. Uncertainty was associated with danger for the future by the parents. Faced with uncertainty about their child’s long-term prognosis, the parents endeavored to find certainty, adopting various strategies to adapt to the unpredictable nature of the situation [10,11]. These findings align with the results of a meta-synthesis of qualitative research on the impact of uncertain clinical relevance results from population genetic screening. The meta-synthesis suggests that results of this nature, not conforming to a common medical model, are rather perceived as ‘full-blown’ diagnoses [20]. Health professionals may characterize screening results as uncertain, but parents frequently interpret them as a diagnosis, and the experience of receiving any form of diagnosis can be traumatic [20].

Another point of interest from the conducted studies is that the negative impact persists over time as the recruited children across studies were up to 12 years old [7]. Parental stress may decrease over time as parents observe the well-being of children with CRMS/CFSPID designation. However, persistent stress may arise due to ongoing medical surveillance. Children, when raised by over-protective parents, may adopt a perception of not being healthy. CF health professionals should conduct family psychological screenings, involving psychologists as needed. While monitoring is necessary, over-medicalization should be avoided. As medical knowledge about CRMS/CFSPID grows, professionals can offer more reassurance over time, reducing parental uncertainty.

CF centers responsible for children with a CRMS/CFSPID designation should take into account the fact that parents often interpret it as a diagnosis. Health professionals should consider implementing psychological screening for both these children and their families, similar to the practice for children with CF. Notably, parents of children with inconclusive CF tend to envision them as normal in the future, picturing them as healthy children without CF [7]. This shift in focus to their children’s health status is evident in the first group of parents. In contrast, parents of healthy controls primarily concentrate on various dimensions of the future, such as academic achievement. It is therefore apparent that the first group of parents alters their focus regarding their child’s development, shifting from general issues to the physical health status. This attitude may also have long-term effects on their child’s development.

Recognizing the psychological burden that conveys this designation for the parents, an updated guidance of the European CF Society Neonatal Screening Working Group provided guidelines of how a CRMS/CFSPID designation should be communicated to parents underlining the importance of this process [2]. They emphasized that this should be acknowledged as a challenging situation, given that the long-term outcomes are not yet known. However, it should be communicated that infants with CRMS/CFSPID are generally well, and the majority of them remain healthy. It is crucial to note that the results of this designation should not be presented as ‘good news’ in comparison to a CF diagnosis. It should be understood that parents of newborns typically do not anticipate a CF diagnosis when providing consent for routine, and often mandatory, NBS for CF. Health professionals must consistently communicate the prognosis of the CRMS/CFSPID designation authentically. Additionally, they should inform primary care physicians about the implications of this designation, educate them on symptoms warranting attention and guide them on when to seek consultation with a CF physician. Adherence to guidelines for managing these children, including communication recommendations, is essential. A recent study indicated that CF health professionals in Europe and the USA were generally aware of the guidelines, reporting only minor deviations in practice.

The studies presented in this review have acknowledged certain limitations as reported by the respective researchers. The often small sample sizes lacked the power to consistently detect significant differences among study groups. The absence of detected differences in psychological burden between the CF and CRMS/CFSPID groups in some studies, as well as the lack of distinctions in parental stress between the CRMS/CFSPID groups and healthy controls in other studies, can be attributed to these limited sample sizes. Additionally, a healthy control group of families with screen-negative children was not always available. Since each study was conducted in the same geographical area, the sample might not be representative enough of the entire population of children having CRMS/CFSPID. Another limitation arises from the variable age range of children among participating families, as well as the variable time of evaluation since the inconclusive CF diagnosis was declared.

Future longitudinal research should be conducted, incorporating a larger number of subjects in each group to discern meaningful yet subtle differences among the groups. A longitudinal design will enable the evaluation of the evolution of psychological burden over time. Furthermore, future studies should ideally be multi-center studies and include participants from different geographical areas, given that stress is influenced by cultural factors. In addition, given that some studies have indicated differences between mothers and fathers when both parents participated, it is recommended that future studies involve the participation of both parents.

Given these limitations, further multi-center studies are warranted to comprehensively explore both short-term and long-term psychological impacts on parents of children with CRMS/CFSPID. Despite these challenges, existing studies have underscored that parents experience emotional disturbances when informed about a CRMS/CFSPID designation. Hence, clear communication between the CF team and the responsible pediatrician is vital to partially alleviate the uncertainty they feel. Additionally, periodic psychological health screening and support appear to be appropriate measures.

## Figures and Tables

**Table 1:** Main characteristics of the presented studies.

	Author/Year/Title	Country	Type of Study	Groups of Parents	Number of Families
1	Perobelli S et al., 2009 [5]	Italy	Quantitative	CRMS/CFSPID- CF Healthy controls	11–11 11
2	Hayeems RZ et al., 2017 [6]	Canada	Quantitative Qualitative	CRMS/CFSPID- CF Healthy controls CRMS/CFSPID	19–14 410 18
3	Tluczek A et al., 2019 [7]	USA	Quantitative Qualitative	CRMS/CFSPID- CF Healthy controls CRMS/CFSPID- CF Healthy controls	20–50 40 20–50 40
4	Ginsburg DK et al., 2023 [8]	USA	Quantitative	CRMS/CFSPID- CF	58–51 (only mothers)
5	Tosco A et al., 2023 [9]	Italy	Quantitative Qualitative	CRMS/CFSPID- CF CRMS/CFSPID- CF	16–16 16–16
6	Tluczek A et al., 2010 [10]	USA	Qualitative	CRMS/CFSPID	10 parents
7	Johnson F et al., 2019 [11]	UK	Qualitative	CRMS/CFSPID	3 couples-2 mothers
